# Physicochemical, Mineralogical, and Mechanical Properties of Calcium Aluminate Cement Concrete Exposed to Elevated Temperatures

**DOI:** 10.3390/ma14143855

**Published:** 2021-07-09

**Authors:** Amirmohamad Abolhasani, Bijan Samali, Fatemeh Aslani

**Affiliations:** 1 Department of Civil Engineering, Babol Noshirvani University of Technology, Babol 47148-71167, Iran; amirmabolhasani@nit.ac.ir (A.A.); Fatemeh.aslani.gh@nit.ac.ir (F.A.); 2 Centre for Infrastructure Engineering, School of Engineering, Western Sydney University, Sydney, NSW 1000, Australia

**Keywords:** calcium aluminate cement, high temperatures, mechanical property, microstructure

## Abstract

One commonly used cement type for thermal applications is CAC containing 38–40% alumina, although the postheated behavior of this cement subjected to elevated temperature has not been studied yet. Here, through extensive experimentation, the postheated mineralogical and physicochemical features of calcium aluminate cement concrete (CACC) were examined via DTA/TGA, X-ray diffraction (XRD), and scanning electron microscopy (SEM) imaging and the variation in the concrete physical features and the compressive strength deterioration with temperature rise were examined through ultrasonic pulse velocity (UPV) values. In addition, other mechanical features that were addressed were the residual tensile strength and elastic modulus. According to the XRD test results, with the temperature rise, the dehydration of the $C_{3}AH_{6}$ structure occurred, which, in turn, led to the crystallization of the monocalcium dialuminate ($CA_{2}$) and alumina ($Al_{2}O_{3}$) structures. The SEM images indicated specific variations in morphology that corresponded to concrete deterioration due to heat.

## 1. Introduction

Building materials such as cement and concrete play a key in construction sustainability, and that is why numerous studies [1,2,3,4] have been conducted in this regard so as to improve the construction quality. A major risk to structures during their lifetime is fire. Drastic physicochemical changes occur in concrete as a result of fire [5,6,7], which lead to concrete degradation and deterioration. As a result, strength loss, cracking, and spalling occur in concrete. In addition, exposure to heat gradually degrades the aggregate–cement paste bonding [2], which leads to weak structural behavior. Guidelines proposed by building codes should be followed to provide a safe thermal performance and ensure safe structural behavior against fire [3]. Thus, the thermal features of concrete exposed to heat are highly important for assessing the performance of various concrete structures against fire. To assess the fire resistance of concrete structures, the material features of interest include the mechanical, thermal, and deformation features, as well as material-specific features consisting of mass loss and spalling induced by fire [4]. Thermal material features with significant effects on the thermal performance of concrete structures include the thermal conductivity, expansion, and diffusivity, as well as the specific heat. Moreover, important mechanical features in this regard include the strength, elastic modulus, and deformation, in that they considerably affect the thermal performance of structural systems [5,6,8,9].

Reviewing the literature indicates that increasing the temperature lowers the mechanical features of mortar, hardened cement paste, and concrete specimens [10,11,12]. As reported by Dias et al. [11], the elastic modulus and strength of the hardened cement paste specimens significantly declined after experiencing temperatures higher than 300 °C. Chan et al. [13] studied the compressive strength of normal- and high-strength concrete specimens after exposure to temperatures up to 800 °C and observed that the compressive strength of both specimens decreased by more than 60%. Sakr et al. [14] observed an inverse relationship of the exposure duration with the mechanical features of concretes.

Among non-Portland specific cement types, calcium aluminate cement (CAC) plays a significant role and can be a proper substitution for a fraction or the whole Portland cement volume. In situations requiring mortar or concrete to show specific properties (such as need for quick hardening, resistance to chemical attacks (particularly acid attacks)), the use of this cement is justified. Several aluminates with different volumes ranging from 40 to 90% make this cement [1]. At present, it has found many, diverse applications in various areas. In this regard, it is used to provide insulation against heat and temperature variation [2], resistance against abrasion and scour in dam spillways and surface coatings [3], resistance against chemical attack (especially acid attack) [4], and ultrafast hardening of concretes.

Khaliq and Khan [15] explored the postheating mechanical features of CACC (containing 70% Al_2_O_3_) and compared the postfire behaviors of this concrete and conventional concrete. It was reported that the alumina existing in the CACC considerably improved the mechanical features in comparison with conventional concrete. In this regard, the residual strength of the CACC was higher compared with that of the conventional concrete, especially for temperatures above 400 °C. It has been established that the mechanical features of this concrete are dependent upon the alumina content.

Therefore, exploring the postfire mechanical properties of CACs with different alumina contents is also required. One of the most common CACs is the one with 40% alumina that is produced by the Fondo factory, Isfahan, Iran, under the commercial name Iran Refectory Cement Co. Several studies have been conducted by the authors regarding the mechanical and fracture properties of calcium aluminate cement containing 38% alumina at the ambient temperature [16,17].

In the present research, the impact of changes in the microstructure of CACC after experiencing heat and on the mechanical features of this concrete was explored. Data about this subject are scarce in the literature. The impact of water-to-cement ratio on the mechanical features of CACC was explored in the previous work of the authors [18], where the proper feature of this concrete at w/c = 0.4 was observed. Therefore, the mix with this w/c ratio was also regarded as the standard mix in this work. Afterward, the postfire performance of CACC specimens was examined in terms of four mechanical features: the residual compressive strength, tensile strength, elastic modulus, and ultrasonic pulse velocity (UPV).

In addition, the heat-induced chemical processes leading to the deterioration and degradation of the cementitious matrix were explored via thermogravimetric analysis (TGA), X-ray diffraction (XRD), scanning electron microscopy (SEM), and energy-dispersive spectroscopy (EDS) methods. Moreover, to obtain a better understanding of the postfire performance of CACC, the observed results for this concrete were compared with the results reported in the literature for other concrete types, especially with the results of Khaliq et al. [15].

## 2. Materials and Methods

### 2.1. Materials

**Figure 1:**
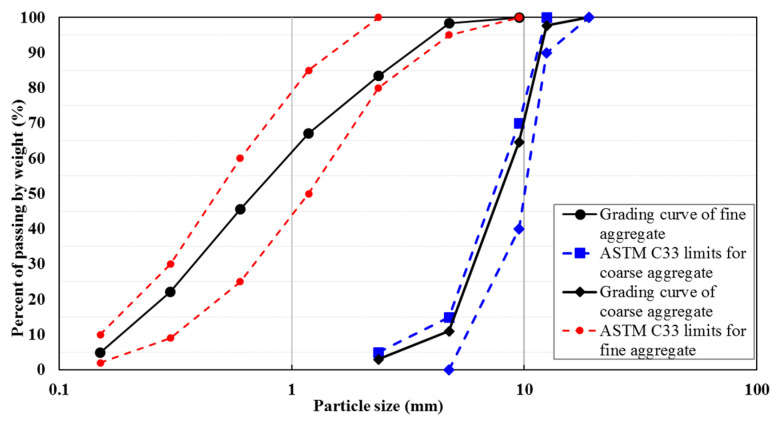
Gradation curves of fine and coarse aggregates.

Here, refractory cement, commercially available under the name IRC-40 (Fondu), made by Iran Refractory Cement Co., Isfahan province, Iran, was used. Calcium aluminate cement was used with a 24 h mechanical strength of 45 MPa and an initial setting time of 200 min. Both coarse and fine aggregates were utilized. Washed river sand was used as the fine aggregate, with a specific gravity of 2.6, fineness modulus of 2.6, and water absorption of 0.7%. The coarse aggregate had a specific gravity of 2.5, a maximum particle diameter of 12 mm, and water absorption of 0.5%. Water used to make the mixtures was tap water. The gradation curves of coarse and fine particles obtained by passing them through a series of sieves in accordance with ASTM C33 [19] are given in Figure 1.

**Figure 2:**
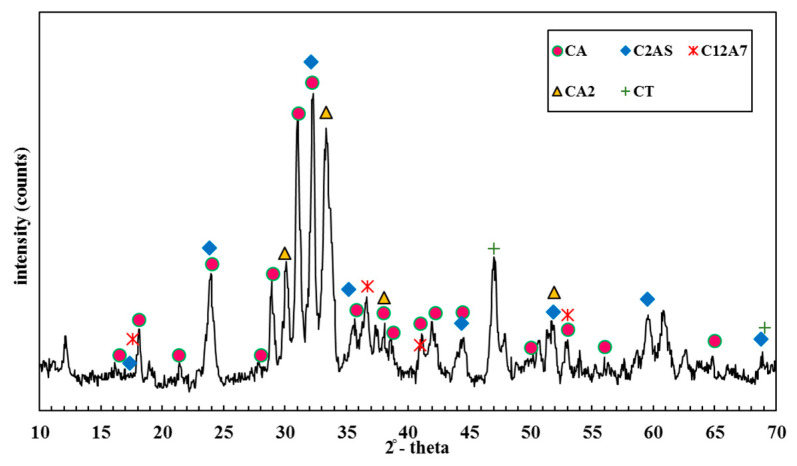
XRD patterns of calcium aluminate cement.

For enhancing the workability of the concrete mix, a superplasticizer based on polycarboxylate ether was employed, which had a specific gravity equal to 1.11. The XRD patterns of CAC are seen in Figure 2. In CAC, the major present phase was CA ($Al_{2}CaO_{4}$), with small quantities of $C_{2}AS$ ($Ca_{2}Al_{2}SiO_{7}$), CT ($CaTiO_{3}$), and $C_{12}A_{7}$(*Ca*_12_*Al*_14_*O*_33_) also detected.

The X-ray fluorescence (XRF) spectroscopy was used to obtain the oxide composition of CAC, with the corresponding results shown in Table 1. A proper correlation is seen with the results reported in [15,20,21].

### 2.2. Mixture Design

The mix proportion of CACC is shown in Table 2. To prepare the concrete mixes, first, the aggregates and powder materials were blended in the dry state for 1 min. Then, water mixed with the superplasticizer was added to the initial mix in several stages, and the mixing continued for 3–5 min. After the complete mixing of the materials, the slump test was conducted on the fresh concrete in accordance with ASTM 143 [22] to evaluate the rheological properties of the CACC. Considering that in the previous works [18], the effect of the water-to-cement ratio on the mechanical and fracture properties of CACC was addressed, the water-to-cement ratio of 0.4 was considered in this work given the better mechanical and fracture behavior it provides for the concrete. Note that the slump test was conducted on all the mix designs, and to obtain the allowable slump values based on the standards, the amount of the superplasticizer was determined.

### 2.3. Preparation of Test Specimens and Test Set-Up

**Figure 3:**
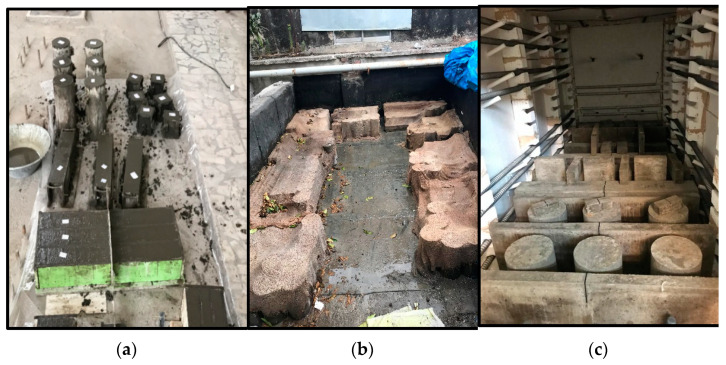
View of the specimens: (**a**) casting and fabrication of fresh concrete; (**b**) curing with a wet sack; (**c**) specimens in the high-temperature furnace.

Standard concrete cylinders with a height and diameter of 300 mm and 150 mm, respectively, were manufactured to assess the elastic modulus and tensile strength based on ASTM C496 [23] and ASTM C469 [24], respectively. In addition, to investigate the compressive strength, three standard concrete cubes with an edge length of 100 mm were manufactured in accordance with BS EN 12390 standard [25] for mix design and then cured in the laboratory environment. The specimens were kept wet by sacking to minimize shrinkage and cured at ambient conditions, namely a temperature of 25 °C and relative humidity of 75%, until the day of the testing (Figure 3).

**Figure 4:**
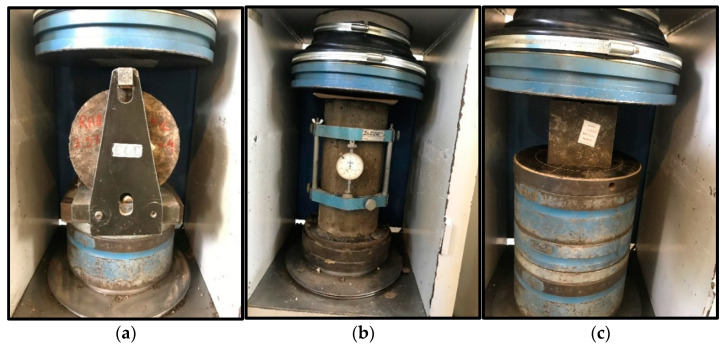
Set-up of hardened concrete tests: (**a**) splitting tensile strength, (**b**) modulus of elasticity, and (**c**) compressive strength.

For three specimens made from each mix design, the average of the results was reported. The tests for measuring the compressive strength, tensile strength, and elastic modulus were conducted with a loading rate of 3, 2.1, and 5.2 kN/s, respectively, applying an automatic testing machine (ELE international) with a load control strategy. Moreover, three beams were tested through the three-point bending test with the dimensions of 500 × 100 × 100 mm^3^ in accordance with ASTM C78M [26]. Figure 4 shows the test samples. The tests were performed under displacement-controlled strategy at a rate of 0.01 mm/s using a closed-loop servo electro-controlled universal testing machine equipped with a load cell of 400 kN capacity and two vertical LVDTs located in the middle of the specimens.

An ultrasonic nondestructive electronic machine was used to measure the UPV, complying with ASTM C597 [27]. A transducer with an oscillation frequency of 54 kHz, transit time accuracy of ±1%, and distance accuracy of ±2% was used. Five points were tested on each specimen by changing the location of transducers on the opposite sides of the specimen, the average of which was reported as a result. Once the transmission time test results were determined, the ultrasonic pulse transmission speed in concrete was obtained by dividing the transmission distance (distance between the measuring points) by the transmission time.

### 2.4. Testing and Heating Procedure

**Figure 5:**
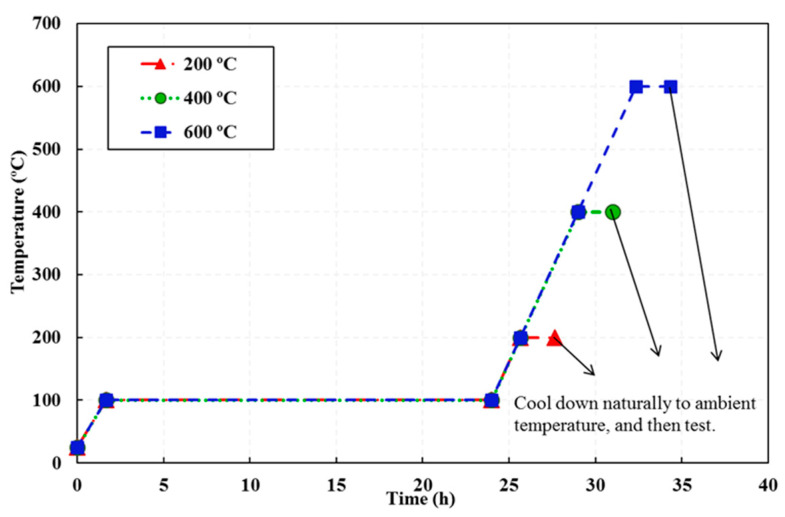
Heating and cooling curves.

The fresh CACC specimens were cured at 25±5 °C for 28 days. In order to minimize the risk of spalling, eliminate capillary water, and prevent spalling, 100 °C was selected as the preheating temperature [28,29] and the specimens were predried at this temperature for 24 h. Afterward, the specimens were heated at three different temperature cycles up to 200, 400, and 600 °C. The first part of each temperature cycle included heating with a rate of 1 °C/min to reach the target temperature, after which the temperature was kept at the target temperature for 2 h to ensure a uniform temperature distribution throughout the specimens. The final part of this cycle included cooling down to reach the ambient temperature. After two hours of exposure to the target temperature, the specimens were not directly exposed to the ambient temperature, and instead, by opening one of the hatches of the furnace, the specimens and furnace were cooled simultaneously. The heating rate was chosen in accordance with the RILEM TC-129 recommendations [30]. For thermal loading at 200 °C and higher, an electric furnace was employed, with the heating regime shown in Figure 5.

Based on the literature, there are three approaches for evaluating concrete strength at high temperatures, which are called stressed testing, unstressed testing, and unstressed residual strength testing. In the stressed testing approach, the specimens are loaded before heating, and this loading continues during the heating phase. When the specimens reach the target temperature, the load/strain is increased up to the failure of the specimens. In the unstressed testing approach, specimens are heated without a preload to reach the target temperature and then tested at this temperature. In the unstressed residual strength testing approach, the specimens are heated without a preload to reach the target temperature and then left to cool to reach the room temperature at which the load/strain is applied to the specimens until failure. In this research, the unstressed residual strength testing approach was employed since it provides the best results for evaluating the postfire properties of concretes [31].

## 3. Results and Discussion

### 3.1. Microscopic Assessment of CACC at High Temperatures

#### 3.1.1. X-ray Diffraction (XRD)

**Figure 6:**
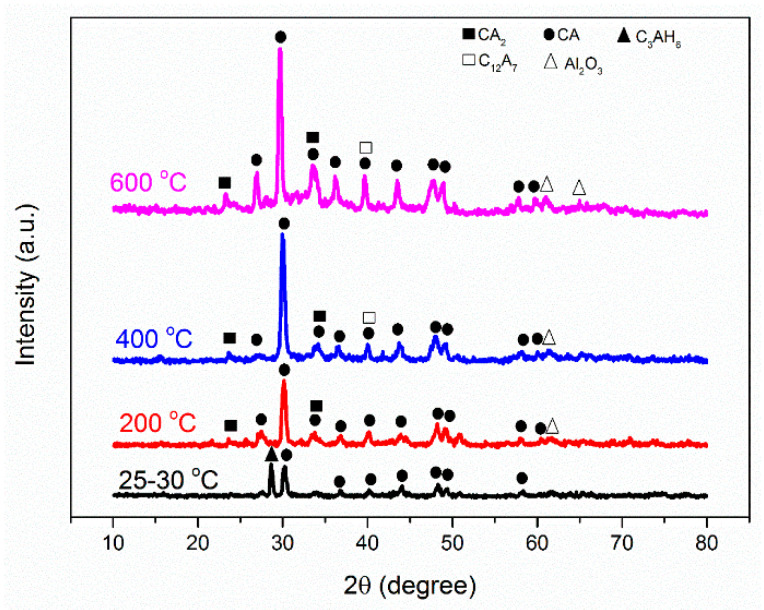
X-ray diffraction pattern of CACC at different temperatures.

In order to investigate the structure of the produced concretes, the X-ray diffraction (XRD) test was used, with the results shown in Figure 6.

According to Figure 6, at ambient temperature, in addition to the peak associated with the monocalcium aluminate (CA), the peak associated with the hydrogarnet ($C_{3}AH_{6}$) can be also seen in the diffraction patterns of the concrete, suggesting the existence of a hydrated structure at ambient temperature. The presence of this structure at ambient temperature as a stable phase in CACCs has previously been established in similar studies [32,33]. As the temperature increases up to 200 °C, the peak related to $C_{3}AH_{6}$ is seen to completely vanish. However, the peak related to the monocalcium dialuminate ($CA_{2}$) as well as alumina ($Al_{2}O_{3}$) is instead become visible. In fact, with increasing temperature, the structure of $C_{3}AH_{6}$ is dehydrated and the alumina and monocalcium dialuminate structures are crystallized in return. The chemical reaction of the hydrogarnet decomposition is as follows (Equation (1)):

(1) $$C_{3}AH_{6}+3CO_{2}\;➔\;3CaCO_{3}+Al_{2}O_{3}+6H_{2}O$$

Moreover, with increasing temperature, the peak located at an angle of around 2θ = 40.17° related to the CA structure moves to lower angles, such that at 600 °C, this peak is seen at an angle of 2θ = 39.72°. This change can be due to the overlapping of the peaks associated with the monocalcium aluminate structure and the $C_{12}A_{7}$ structure. This overlapping is attributed to the fact that the angle associated with this structure is close to and lower than that of the peak of the CA structure, which in turn leads to the movement of this peak. The conversion reaction of hydrogarnet to $C_{12}A_{7}$ is as follows:

(2) $$4\;C_{3}AH_{6}+3AH_{3}\;➔\;C_{12}A_{7}+33H$$

In addition, as the temperature increases, it is seen in this structure that the intensity of the peaks increases, due to the increase in the crystallite degree of crystalline structures in the concrete.

#### 3.1.2. FE-SEM and EDS Analysis

**Figure 7:**
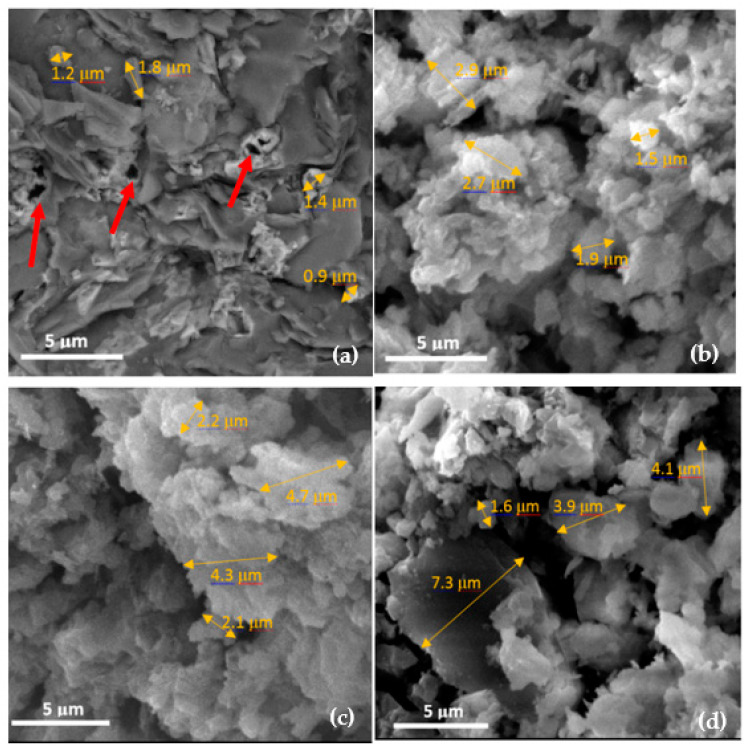
SEM images of the CACC specimens at (**a**) ambient temperature, (**b**) 200 °C, (**c**) 400 °C, and (**d**) 600 °C.

Figure 7 presents the results of the scanning electron microscopy (SEM) test on the CACC specimens exposed to different temperatures.

As Figure 7 shows, the morphology of the CACC particles drastically changes by increasing the temperature from the ambient temperature to 600 °C. By comparing the images of the surface morphology of the concretes at ambient temperature (Figure 7a) and 200 °C (Figure 7b), it is seen that structure of the nanoparticles changes from an almost compact state with small pores (red arrows) at ambient temperature to a granular state with larger pores at 200 °C. The mean size of the particles seen in Figure 7a was obtained as approximately 1.3 µm for the nonheated specimen, while for the specimen exposed to 200 °C (Figure 7b), it was obtained as approximately 2.2 µm. Moreover, the pore size in the nonheated specimen was around 0.9 µm, while it was around 1.4 µm in the specimen heated to 200 °C. In fact, as the particle size increased with the temperature rise, the voids among the particles also increased, indicating an increase in the number and size of pores in the concrete structure. This increasing trend of particle size with temperature rise continued, and at 400 and 600 °C, the mean particle size reached 3.3 µm and 4.2 µm, respectively. The greatest pore size among these specimens pertained to the one heated at 600 °C, for which the mean pore size was around 2.5 µm.

As can be seen in Figure 7, as the particle sizes and thus the size of voids among the particles increase with temperature, the ductility and mechanical properties of the concrete are affected. K. Hossain [34] reported that the strength reduction at high temperatures resulted from the increase in the particle size as well as porosity. When a specimen is subjected to flexural loading, fine pores on the micrometer scale are filled slowly with cement particles, which in turn prevents the sudden rupture of the concrete, rendering the concrete more ductile. The SEM images provided in the paper were taken from the cement matrix particularly in the ITZ after exposure to the target temperature, and the effect of the cement particle size variability on the thermal properties of this concrete was not investigated. The influence of CAC particle size on the thermal properties of interest will be studied in future works. Gu et al. [35] indicated that the volumetric stability and thermal shock resistance of the CAC-bonded castables were improved by replacing unground CAC with ground CAC since the adding of ground CAC produced smaller-sized CA_6_.

**Figure 8:**
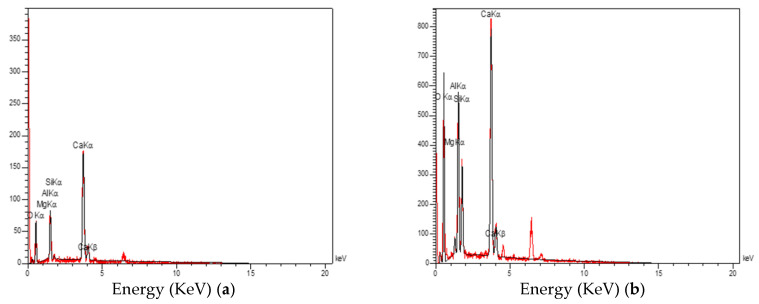

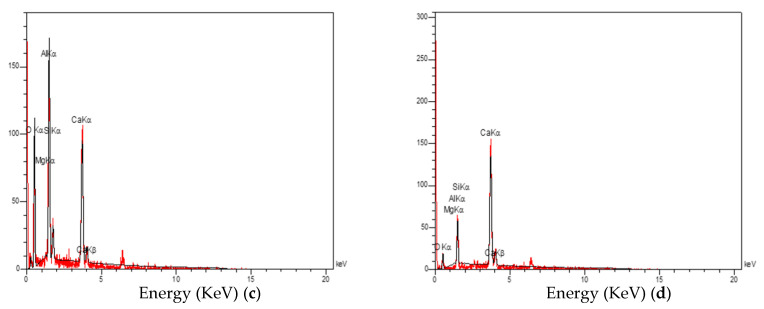
EDS of the CACC specimens at (**a**) ambient temperature, (**b**) 200 °C, (**c**) 400 °C, and (**d**) 600 °C.

The energy-dispersive spectroscopy (EDS) analysis was performed to identify the type and semiquantities of the constituent materials of concretes. The results of this test for the CACC specimens are given in Figure 8.

According to Figure 8, the existence of elements calcium, magnesium, aluminum, silica, and oxygen in the produced concrete structure can be confirmed. The elimination of elements carbon and oxygen with increasing temperature can be due to the decomposition of the calcium carbonate in the concrete structure according to the following reaction [36]:

(3) $$CaCO_{3}\;➔\;CaO+CO_{2}$$

The above reaction shows that the carbon and oxygen in the structure of calcium carbonate are converted to gaseous carbon dioxide and exit the matrix.

#### 3.1.3. Thermogravimetric Analysis

**Figure 9:**
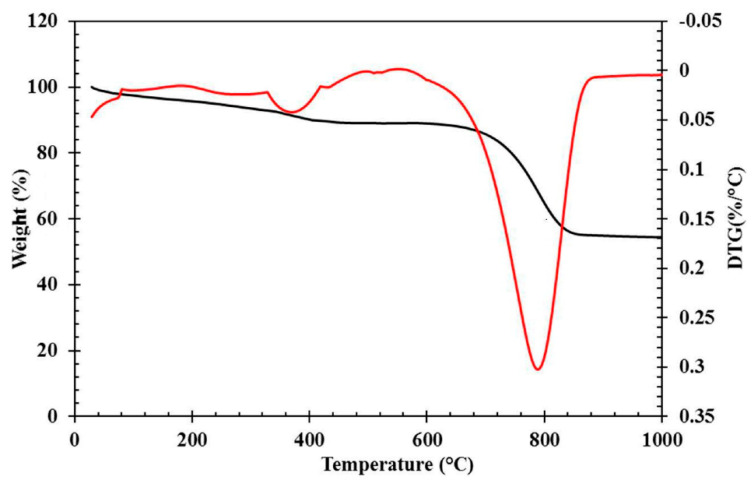
DTG and TGA curves of sample under various curing regimes.

In order to examine the thermal properties of this concrete, the thermogravimetric analysis (TGA) was used, with the obtained results shown in Figure 9.

By analyzing the TGA graph, the behavior of a specimen against the temperature can be investigated. However, sometimes it is needed to investigate the decomposition rate of a material, which is not shown in the graph. As a result, the weight loss rate is shown in the DTGA graph. In Figure 8, the vertical axis on the left is the residual weight percentage and the vertical axis on the right is the weight loss rate. In Figure 9, the weight loss of the CACC at temperatures lower than 130 °C is due to the evaporation of water in the structure of this concrete [37]. The 8% weight loss of the CAC concrete in this temperature range shows that 8% of the concrete is composed of physical water. According to Figure 9, the peak in the derivative thermogravimetric (DTG) curve of the CAC specimen at around 370 °C is attributed in similar studies to the elimination of hydrogarnet [37]. The severe weight loss of the CAC specimen at a temperature of around 800 °C is associated with the decomposition of the calcium carbonate in the concrete matrix, as reported by Kumar et al. [38]. It is observed that this weight loss is around 33% for the CAC specimen. The presence of compounds containing calcium was previously seen in the XRD results, which is also verified by the TGA results. In general, as temperature increases from the ambient temperature to 1000 °C, a total of 45.57% of the weight of the specimen is lost. This finding is attributed to the elimination of the chemical water together with the carbon and oxygen in the structure of the compounds found in the concrete. This reduction in the carbon and oxygen elements was also observed in the energy-dispersive spectroscopy (EDS) results, which is verified here.

### 3.2. Effect of Curing Regimes on the Mechanical Properties of CACC

#### 3.2.1. Compressive Strength

**Figure 10:**
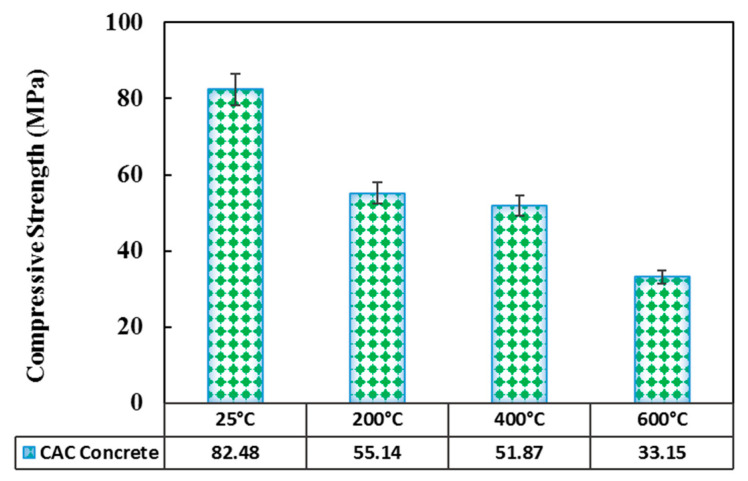
Compressive strength of concrete as a function of temperature.

Figure 10 reports the cubic compressive strength values of the concrete specimens exposed to different temperatures. It shows the decline of the compressive strength with the temperature rise, which correlates well with the literature. Bamonte and Gambarova [39] reported that in the normal-strength self-compacted concrete (SCC) exposed to 200, 400, and 600 °C, the compressive strength reductions were 25, 38, and 70%, respectively. Likewise, the corresponding values reported for the high-strength SCC were 20, 42, and 70%, respectively. Fares et al. [40] attributed this decline to the weakened Van der Waals forces between the C-S-H layers, which lowers the C-S-H energy level and forms the silanol (Si-OH:OH-Si) groups, thus leading to the lower bond strength. Furthermore, Phan et al. [41] reported the strength reduction of high-performance concrete (HPC) at 200, 300, and 450 °C to be 20, 34, and 53%, respectively. Peng et al. [42] investigated HPC specimens exposed to high temperatures and noted that as the temperature increased from 25 to 800 °C, the compressive strength declined from 97.3 to 24.9 MPa.

In the present study (Figure 10), the compressive strength decreased from 82.48 MPa at 25 °C to 55.14 MPa at 200 °C, indicating a 33% reduction, due to the evaporation of the physically bound water (dehydration of C_3_AH_6_).

As the temperature increased further up to 400 and 600 °C, the compressive strength experienced a 37 and 60% reduction. Khaliq [43] conducted a comparison between the normal-strength concrete (NSC) and CACC and found that in the temperature range of 200–400 °C, the CACC had better performance and lost only 18% of its compressive strength. In this study, however, this reduction in the same temperature range was around 4%. Bažant et al. [44] reported that at temperatures above 350 °C, all the chemically bound water evaporated and the porosity increased significantly. In addition, the cement hydrates start to decompose, and in the siliceous aggregate, α-quartz is converted to β-quartz with 5% volume expansion, resulting in a drastic reduction in the concrete compressive strength.

#### 3.2.2. Splitting Tensile Strength

**Figure 11:**
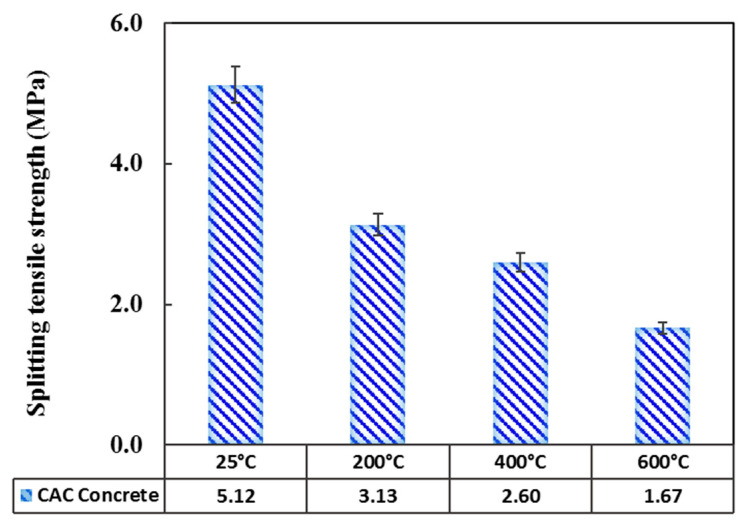
Splitting tensile strength of concrete as a function of temperature.

The ultimate tensile strength of the cylindrical specimens exposed to heat was measured and recorded after cooling, with the associated results given in Figure 11. The tensile strength declined with increasing temperature, which is in line with the literature.

In this regard, Sakr [14] investigated high-weight concrete (HWC) specimens and stated that the tensile strength decreased from 2.55 MPa at the ambient temperature to 1.77, 0.78, and 0 MPa at 250, 500, and 750 °C, respectively. Zhang and Bicanic [45] addressed the high-performance concrete (HPC) exposed to temperatures of 25, 150, 250, 350, and 450 °C and reported the tensile strength values at these temperatures to be 4.47, 3.61, 3.31, 2.91, and 2.48 MPa, respectively. Other researchers focused on the SCC and found that after exposure to temperatures of 200, 400, and 600 °C, the residual tensile strength was 79, 59, and 39%, respectively, of the reference value. Li et al. [46] reported a similar decreasing trend by investigating the concrete containing ground granulated blast furnace slag (GGBFS). In the studies of Khaliq and Kodur [47], Siddique and Kaur [48], and Zhao and Gao [49] on HSC and Ge et al. [50] on fiber-reinforced HPC, this similar decreasing trend was also observed.

In the present research, as the temperature was raised to 200 and 400 °C, the tensile strength declined by around 39 and 50%, respectively (Figure 11). A strength reduction of around 26% was reported when the temperature was increased to 400 °C. This difference between the results of the present study and Khaliq’s study is a result of the different heating rates in the two studies, as previously mentioned. This tensile strength decline is a result of the concrete microstructure dehydration caused by the increased thermal stresses [51,52]. As the exposure temperature was raised to 600 °C, small and big cracks appeared, which led to an 18% of reduction in the tensile strength compared to 400 °C. This is a result of the different thermal expansion rates of the aggregate and cement paste as well as the decomposition of the calcium hydroxide in the cement paste.

#### 3.2.3. Elastic Modulus

ASTM C469 [24] proposed Equation (4) to obtain the elastic modulus, E, in which S_1_ is the stress related to $\varepsilon_{1}$, S_2_ is the strength associated with 40% of the ultimate load, $\varepsilon_{1}$ is equivalent to a strain of 0.005, and $\varepsilon_{2}$ is the longitudinal strain corresponding to the stress S_2_.

(4) $$E=\frac{S_{2}-S_{1}}{\varepsilon_{2}-\varepsilon_{1}}$$

**Figure 12:**
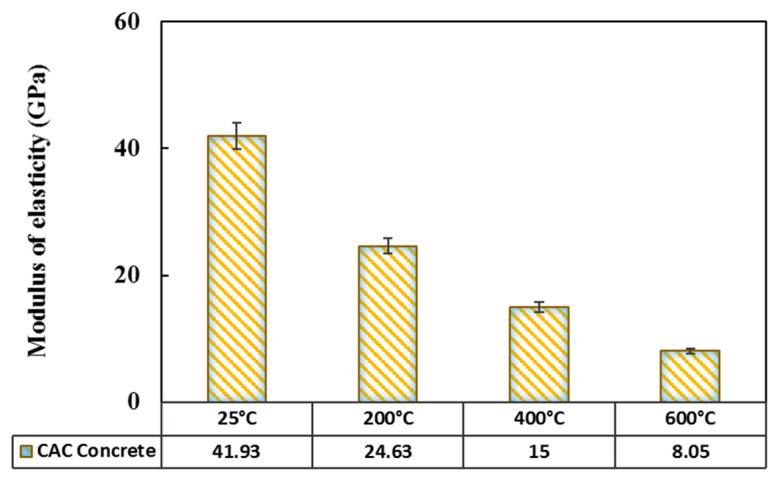
Modulus of elasticity of concrete as a function of temperature.

Figure 12 shows the elastic modulus values of the cooled cylindrical specimens after exposure to different temperatures. The values obtained here have a good consistency with those reported in the literature. In studies on HSC specimens by Phan and Carino [53] and HPC specimens by Phan et al. [54], a similar decreasing trend was found.

In the present study, the elastic modulus decreased from 41.93 GPa at the ambient temperature to 24.63 GPa after exposure to 200 °C, showing a 41% reduction, as can be seen in Figure 12. The initial reduction in the elastic modulus of the CACC specimens is attributed to the conversion of the hydrates in the concrete microstructure. This leads to a higher porosity and a significant loss of the chemically bound water due to this conversion reaction, as discussed extensively in the XRD and SEM sections. Khaliq and Khan [15] reported that the modulus reduction was greater in the CAC concrete up to 300 °C. However, at higher temperatures, the rate of modulus reduction in the CAC concrete became lower than that in the conventional concrete.

As the temperature further increased to 400 and 600 °C, the obtained elastic modulus values were 15 and 8.05 GPa, respectively, indicating a drastic reduction. In this regard, Fares et al. [40] reported that after 450 °C, the modulus decreased significantly. This is due to the fact that at this temperature, these specimens become very brittle, and numerous cracks appear on their surface. Concerning the XRD results, this drastic modulus reduction after the exposure to 200 and 400 °C can be attributed to the conversion of the hydrogarnet leading to the decomposition of hydration products. Khaliq and Khan [15] compared the residual moduli of the conventional and CAC concretes and found that the residual modulus was higher in the CAC concrete. This difference was attributed to the higher quality and more compact microstructure of the CAC concrete compared to the conventional concrete (due to a greater content of alumina).

#### 3.2.4. Ultrasonic Pulse Velocity of Test Specimens

**Figure 13:**
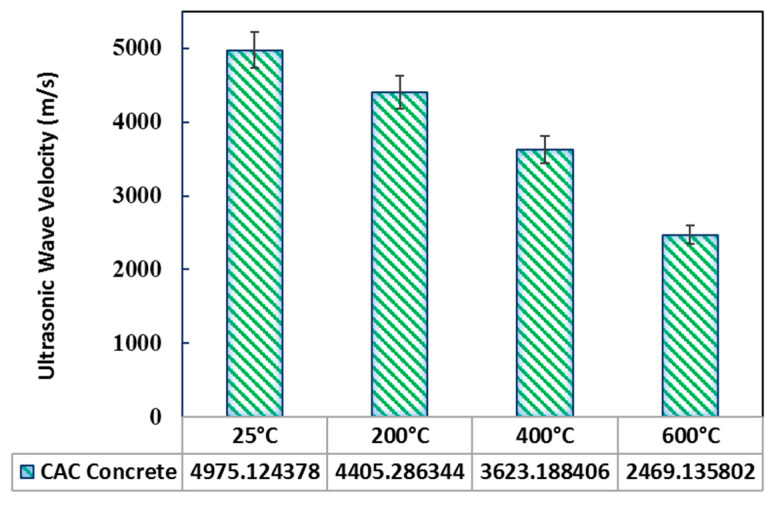
Ultrasonic wave velocity of concrete as a function of temperature.

Ultrasonic wave velocity is a nondestructive method for measuring the velocity of sound in the materials considering that the velocity of a wave is a function of the porosity inside the material. The proper relation between the mechanical properties of the concrete and the velocity of the ultrasonic waves can be determined, and many of the mechanical properties, as well as the quality, of the concrete can be evaluated nondestructively. It is observed that before exposure to different elevated temperatures, UPV values in specimens vary from 4700 to 5000 m/s, indicating the good condition of concrete at the ambient temperature. This is because an ultrasonic pulse velocity higher than 4.575 km/s generally represents the excellent quality of concrete [55]. The excellent ultrasonic pulse velocity was attained mostly due to the improved pore structure of concretes with reduced porosity and small pore sizes. Figure 13 reveals the change in ultrasonic pulse velocity (UPV) of concrete exposed to various temperatures. It can be seen that during the initial temperature, the change in velocity was high, and as the temperature increased, the velocity decreased. It is generally understood that this reduction in the change of velocity is due to the deterioration of the microstructure of the concrete exposed to high temperatures. Topcu and Demir [56] attributed this type of change to degradation of the C-S-H gel at a temperature above 450 °C which increases the number of air voids in the concrete, decreasing the transmission speed of sound waves through the test specimens.

#### 3.2.5. Comparison between Mechanical Properties

**Figure 14:**
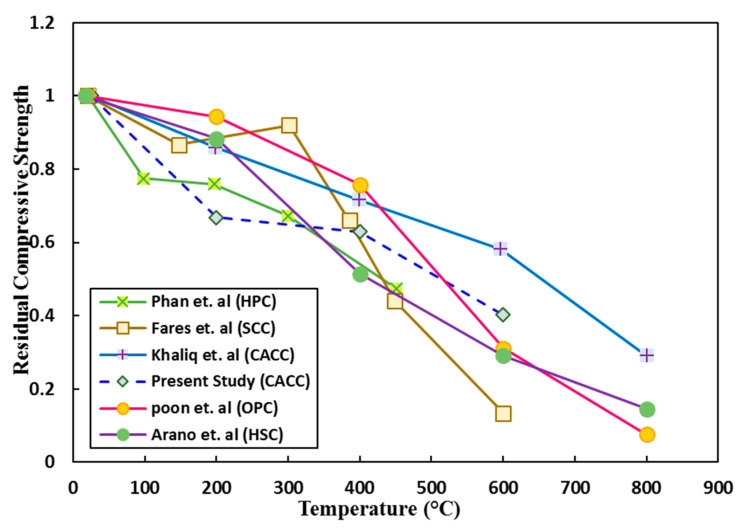
Concrete residual compressive strengths at different heating temperatures.

Many investigations in recent years have focused on the thermal performance of different concretes. Note that regarding the thermal performance, in addition to common variables considered in different investigations under normal conditions, variables including the heating rate and time of exposure to the target temperature are factors notably affecting the microstructure and mechanical properties of concretes. In this regard, as the heating rate decreases and the time of exposure increases, the deterioration of the microstructure and mechanical parameters is exacerbated. Due to the importance of the different performances of various concretes against high temperatures, in this section, an attempt is made to provide a comparison between the residual mechanical properties of CACC with those of other concretes (investigated in the literature) after exposure to heat, with some parameters being variable. By comparing the residual compressive strength of different concretes shown in Figure 14 [15,40,41,54,57], the better performance of CACC, particularly for temperatures higher than 400 °C, is observed; this results from the lower dehydration and chemical water evaporation in the CACC cement matrix relative to OPC. In the results reported by Khaligh, the residual compressive strength percentage is higher than that reported in this study; this is the result of a higher alumina content in the cement and a higher heating rate in that study.

**Figure 15:**
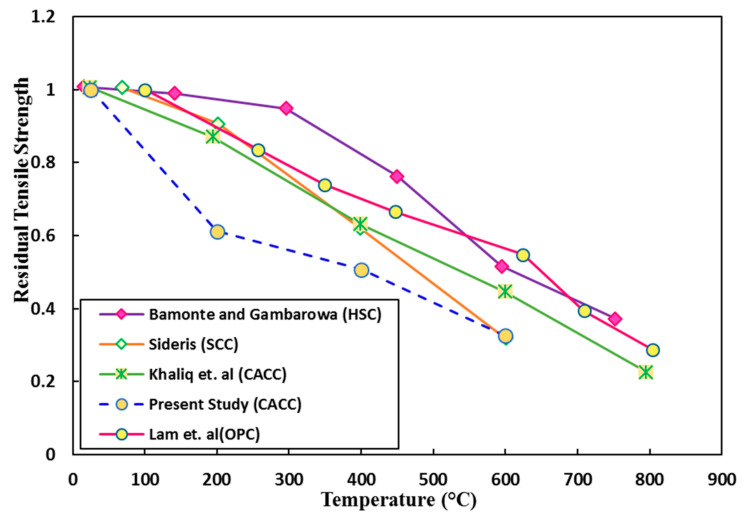
Concrete residual splitting tensile strengths at different heating temperatures.

In Figure 15, the residual tensile strength percentage of CACC in this study is compared with that in other studies, which shows a relatively weaker behavior of CACC compared with other concretes. [15,58,59,60]. In general, increasing the temperature and time of exposure to the target temperature leads to an increase in porosity and permeability (which is also seen in SEM images), which in turn facilitates the transfer of water and water vapor in concrete. However, this dual effect leads to more complexity, in that although higher permeability levels can lower pore pressure and the spalling possibility, they tend to further degrade the tensile strength. This phenomenon contributes to the weak tensile performance of CACs compared with other concretes.

**Figure 16:**
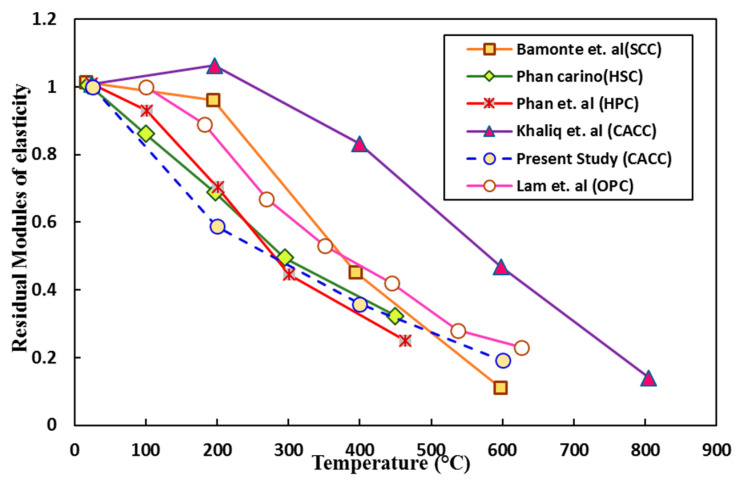
Concrete residual elastic modulus at different heating temperatures.

In addition, Figure 16 [15,39,41,59,61] compares the residual elastic modulus percentage of different concretes with that of the CACC tested in this study and shows the better performance of CACC, particularly for temperatures above 400 °C, similar to the case with the compressive strength. In the study by Khaliq et al. [15], as previously mentioned, due to a higher alumina content of the cement and a higher heating rate, the residual elastic modulus percentages for exposure temperatures of up to 600 °C are higher than the corresponding results reported here.

## 4. Conclusions

The aim of this study was to explore the microstructural changes and their effects on the mechanical and fracture properties of the CACC upon exposure to extreme temperatures of up to 600 °C. The results of this research are beneficial and significant for understanding the microstructural degradation and decomposition of chemical compounds in the cement matrix and the variation in the mechanical and fracture features of this concrete after the fire. The main findings are as follows:

- Weight loss is a measure that can determine the governing process under high temperature ranges. Below 200 °C, the weight loss is merely due to the evaporation of the capillary water, which is a physical process. At 200–400 °C, weight loss is due to the gradual evaporation of the chemical water, and the concrete experiences a chemical–physical process. For temperatures above 400 °C, the weight loss is mainly due to the evaporation of the chemically bound water and decomposition of the compounds, which are chemical processes.
- Based on the results of the XRD and EDS tests, as the temperature increases, the structure of $C_{3}AH_{6}$ is dehydrated, and in return, the alumina ($Al_{2}O_{3}$) and monocalcium dialuminate ($CA_{2}$) structures are crystallized. The SEM images show that the structure of nanoparticles at the ambient temperature was converted from an almost compact state to a granular structure as the diameter of particles, voids, and porosity increased. In this regard, the highest particle size was observed at 600 °C, which led to higher ductility and lower strength.
- The mechanical properties of the concrete specimens (compressive, tensile, and elastic modulus) degraded with the increase in temperature. At 25–200 °C, due to the evaporation of capillary and gel waters, the reduction in the mechanical properties was significant. However, at 200–400 °C, the variation in the properties was not significant. At 400–600 °C, a severe reduction was seen in the mechanical properties, due to the conversion of hydrogarnet and increase in porosity.

## Figures and Tables

**Table 1:** Percentage of oxide composition for CAC.

$SiO_{2}$	$\;Al_{2}O_{3}$	$Fe_{2}O_{3}$	MgO	CaO	$P_{2}O_{5}$	$Na_{2}O$	$K_{2}O$	MnO	Ig. Loss	Blaine Surface Area	Specific Gravity	Reference
5.25	38.22	13.87	0.97	37.49	0.089	0.311	0.072	0.078	1.04	2900	2.98	Present study
4.98	38.23	15.4	0.71	37.53	0.13	0.03	0.23	-	0.02	-	2.94	Adams et al. [20]
3.75	39.5	16	1.5	38	-	0.2	0.21	-	-	3150	3.25	Vafaei et al. [21]

**Table 2:** Mix proportion of CACC.

Cement ($kg/m^{3}$)	Water ($kg/m^{3}$)	Coarse Aggregate ($kg/m^{3}$)	Fine Aggregate ($kg/m^{3}$)	Super Plasticizer ($kg/m^{3}$)	Slump (mm)
450	180	840	905	0.378	100

## Data Availability

Data Sharing is not applicable.
